# Physical Activity in the Prevention of Development and Progression of Kidney Disease in Type 1 Diabetes

**DOI:** 10.1007/s11892-019-1157-y

**Published:** 2019-05-31

**Authors:** Drazenka Pongrac Barlovic, Heidi Tikkanen-Dolenc, Per-Henrik Groop

**Affiliations:** 1 University Medical Center Ljubljana, Ljubljana, Slovenia; 2 Faculty of Medicine, University Ljubljana, Ljubljana, Slovenia; 3 Folkhälsan Institute of Genetics, Folkhälsan Research Center, Biomedicum Helsinki, University of Helsinki, Haartmaninkatu 8, P.O Box 63, FIN-00014 Helsinki, Finland; 4 Abdominal Center Nephrology, University of Helsinki and Helsinki University Central Hospital, Helsinki, Finland; 5 Research Programs Unit, Diabetes and Obesity, University of Helsinki, Helsinki, Finland; 6 Department of Diabetes, Central Clinical School, Monash University, Melbourne, Victoria Australia

**Keywords:** Type 1 diabetes, Exercise, Physical activity, Diabetic kidney disease, Chronic kidney disease

## Abstract

**Purpose of Review:**

Physical activity is a fundamental part of lifestyle management in diabetes care. Although its benefits are very well recognized in the general population and in people with type 2 diabetes, much less is known about the effects of exercise in type 1 diabetes. In particular, exercise effects in relation to diabetic kidney disease (DKD) are understudied. Some uncertainties about physical activity recommendations stem from the fact that strenuous exercise may worsen albuminuria immediately after the activity. However, in middle-aged and older adults without diabetes, observational studies have suggested that physical activity is associated with a decreased risk of rapid kidney function deterioration. In this review, we focus on the role of physical activity in patients with DKD and type 1 diabetes.

**Recent Findings:**

Hereby, we present data that show that in individuals at risk of DKD or with established DKD, regular moderate-to-vigorous physical activity was associated with reduced incidence and progression of DKD, as well as reduced risk of cardiovascular events and mortality.

**Summary:**

Therefore, regular moderate-to-vigorous exercise should become a central part of the management of individuals with type 1 diabetes, in the absence of contraindications and accompanied with all needed educational support for optimal diabetes management.

## Introduction

Patients living with diabetes are recommended to engage in 150 min of moderate-to-vigorous intensity aerobic activity per week to realize benefits on metabolism, fitness, and well-being [1]. Patients are also encouraged to avoid being sedentary for more than 2 consecutive days (Table 1). In addition, resistance and flexibility exercises are recommended two to three times per week [1]. Active adults with type 1 diabetes achieve better blood pressure values and lipid profile and have improved body composition, cardiorespiratory fitness, and well-being compared with their inactive counterparts [4, 5]. Although individuals with regular physical activity have reduced daily insulin dose, its effect on glycemic control, expressed by glycated hemoglobin A_1c_ (HbA_1c_), is not straightforward. The reasons why regular physical activity may not translate directly into lower HbA_1c_ values may be increased calorie intake, decreased insulin dose, and a reduction in glycemic variability [6]. Of note, exercise does not only decrease blood glucose concentration, but has also been postulated that the addition of brief intervals of high intensity, sprint-type exercise to aerobic exercise can minimize the risk of hypoglycemia and even decrease the risk of late nocturnal hypoglycemic episodes [7, 8].

**Table 1:** Recommended physical activity in patients with diabetes [1, 2]

Type	Duration/week	Special notice
Aerobic	150 min	Moderate-to-vigorous intensity*, no more than 2 days without activity
Resistance	2–3 sessions	Sessions should be scheduled on non-consecutive days; sessions should include 8–10 exercises with completion of 1–3 sets of 10–15 repetitions using free weights, resistance machines, resistance bands or performing exercise against body weight
Flexibility	2–3 sessions	Especially recommended for older adults

*Moderate intensity can be defined as the intensity of 3-5,9 MET (metabolic equivalent) or exercise when your breathing is faster but compatible with speaking full sentences; vigorous intensity can be defined as the intensity of ≥ 6 MET or exercise when breathing is very hard and you cannot carry on a conversation comfortably [3]

Unfortunately, persons with type 1 diabetes less often engage in physical activity compared with the general population [9], with a large percentage of individuals not achieving the recommended minimum amount of moderate-to-vigorous aerobic activity per week. In a recent large cross-sectional study of 18,028 adults with type 1 diabetes from Austria and Germany, it was shown that less than 20% of individuals manage to engage in aerobic exercise more than 2 times per week and only about 40% of individuals perform structured exercise [4]. There were many potential barriers to exercise identified (Table 2), with the most important ones being fear of hypoglycemia, loss of glycemic control, and inadequate knowledge of diabetes management around and during exercise [9].

**Table 2:** Perceived barriers to physical activity summarized according to Brazeau et al. [9] and Clarke et al. [10]

Type 1 diabetes	Chronic kidney disease
Fear of hypoglycemia	Poor health
Fear of loss of glycemic control	Fear of injury or aggravating their condition
Inadequate knowledge of diabetes management in relation to exercise	Lack of guidance from their health professional
Work schedule	Lack of facilities
Low levels of fitness	Lack of social support
Lack of social support	

Epidemiological data suggest that around 20% of individuals with type 1 diabetes will develop end-stage renal disease (ESRD) during their lifetime, although the incidence of ESRD is decreasing [11]. The presence of diabetic kidney disease (DKD) is an important risk factor for ESRD and also of cardiovascular or non-cardiovascular mortality, even in patients with an estimated glomerular filtration rate (eGFR) above 60 ml/min/1.73 m^2^ [12–14]. In this review, the possible mechanisms promoting beneficial effects of physical activity on DKD will be summarized. Second, we briefly review data on the role of exercise in non-diabetic kidney disease. Finally, we will describe the basic recommendations for applying the existing knowledge on the effects of physical activity on DKD development and progression in type 1 diabetes into clinical practice.

## Mechanisms of Beneficial Effects of Physical Activity on Chronic Kidney Disease

**Fig. 1:**
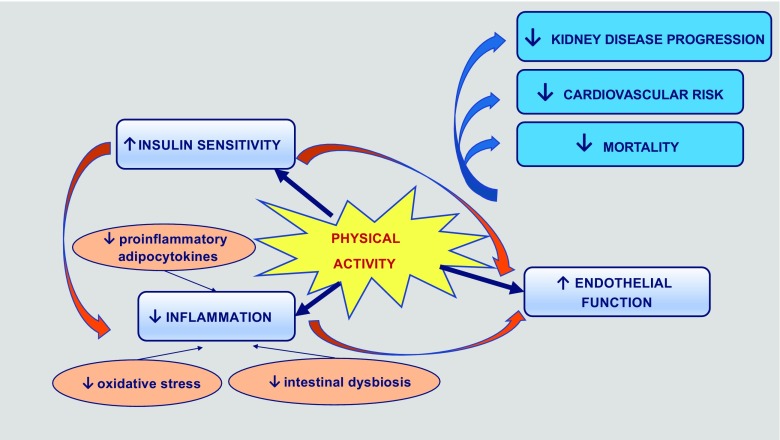
Diagram presenting mechanisms through which exercise influences beneficial outcomes in patients with DKD

Even a short period of structured physical activity triggers many changes at multiple tissue levels that could contribute to its beneficial effects in the prevention of initiation or progression of kidney disease (Fig. 1).

### Physical Activity Improves Endothelial Function

Physical activity modulates nitric oxide (NO) synthesis by increasing NO precursor l-arginine bioavailability and activity of endothelial NO synthase [15]. In addition, it reduces NO degradation by reducing reactive oxygen species (ROS) [15]. Nitric oxide is known as one of the most important paracrine modulators of renal function, effecting renal autoregulation, glomerular filtration, renin secretion, and salt excretion [16]. Deficiency in nitric oxide has been implicated in renal disease deterioration, including worsening of glomerulosclerosis and tubulointerstitial inflammation with fibrosis [17].

### Physical Activity Improves Insulin Sensitivity

Even a single bout of exercise, independent of weight loss, leads to increased insulin sensitivity [18]. Namely, it facilitates non-insulin-mediated glucose uptake into skeletal muscle and activates AMP-activated protein kinase (AMPK). This results in the phosphorylation of the Rab-GTPase-activating protein TBC1D1 (Tre-2/BUB2/cdc 1 domain family, member 1), involved in regulation of intracellular membrane trafficking. Phosphorylation inactivates TBC1D1 thereby enabling GTP to react with Rab proteins on the GLUT4 vesicles, thus, increasing GLUT4 vesicle translocation from cytosol to the cell membrane and increasing glucose uptake into the cell [19]. In this way, exercise increases muscle glucose uptake, independent of insulin. In advanced kidney disease, insulin resistance was shown to be mainly the result of the impaired muscle phosphatidylinositol 3-kinase/Akt signaling contributing to its impaired anabolic response, increased catabolism, and muscle wasting [20, 21]. Also, at earlier stages of kidney disease, insulin resistance leads to hyperinsulinemia that activates the phosphatidylinositol-3-kinase/protein kinase B (Akt) pathway. In endothelial cells, this activation results in the reduction of Akt-dependent synthesis of nitric oxide and in increased mitogen-activated protein kinase (MAPK)-dependent vasoreactivity, contributing to microvascular damage [22]. However, even more interesting is the role of insulin signaling at the level of the podocyte. Of note, podocytes have been shown to be responsive to insulin for glucose uptake and metabolism. In vitro models have demonstrated that podocyte-specific insulin resistance leads to albuminuria, together with thickened glomerular basement membrane and glomerulosclerosis [23]. However, it is yet unknown whether exercise-induced improvement in insulin sensitivity could translate into improved podocyte insulin signaling.

### Physical Activity Induces Changes in Adipose Tissue and Adipocytokines

Visceral adipocytes have been shown to produce an array of adipocytokines that could contribute to endothelial injury in the kidney influencing progression to chronic kidney disease (CKD), such as angiotensinogen, TNF-α, plasminogen activator inhibitor-1, resistin, ghrelin, and leptin [24]. Weight loss protects against progression of CKD [25]. Moreover, altered distribution of adipose tissue, and more specifically, loss of visceral adiposity are associated with a decrease in reactive oxygen species and inflammation that could result in improved renal outcomes [26].

### Physical Activity Reduces Inflammation

CKD is a chronic inflammatory state. Increased production and decreased clearance of pro-inflammatory cytokines, oxidative stress, and acidosis, as well as susceptibility to infections and intestinal dysbiosis, all contribute to chronic inflammation that is adversely associated with albuminuria and measures of kidney function [27, 28]. In addition, pro-inflammatory molecules, such as TNF-α, are potent triggers of osteoclast activation and contribute to the adverse effects on the bone and skeletal muscle metabolism with resultant bone resorption, sarcopenia and cachexia, and also vessel wall diseases [29]. Exercise remains an important tool in reversing the inflammatory processes and their negative consequences [30, 31].

## Physical Activity and Chronic Kidney Disease

The level of physical activity decreases with declining kidney function [32]. Factors associated with CKD contributing to exercise intolerance and serving as meaningful barriers to regular physical activity include anemia, hypertension, and bone and muscle wasting (Table 2) [33]. Patients also listed fear of injury or aggravating their disease, a lack of guidance from healthcare professionals, and a lack of local facilities as additional factors leading to sedentary lifestyle in CKD [10].

Most of the studies addressing exercise in individuals with CKD were short-term and confirmed the beneficial effects of exercise on cardiovascular risk factors. For example, a comprehensive Cochrane review that included 45 randomized controlled studies with 1863 participants with CKD who engaged in different types of physical activity for at least 8 weeks demonstrated that regular exercise has beneficial effects on physical fitness, walking capacity, blood pressure, and heart rate, as well as on quality of life [34, 35]. In addition, observational cohort studies confirmed the beneficial effects of exercise on cardiovascular disease or mortality [36–38] in patients with CKD.

### Benefits of Physical Activity Across the Spectrum of Kidney Disease

During exercise, as the muscle oxygen requirement increases, the blood flow to the internal organs, including the kidneys, is reduced. Renal plasma flow and glomerular filtration rate decrease in response to increase in exercise intensity. Therefore, even though physical activity has been shown to have a positive impact on cardiovascular risk factors, it is important to understand its effects on renal function at different levels of CKD.

Exercise was shown to have beneficial [39, 40] or inconclusive [41] effects on the preservation of kidney function in observational cohort studies. Specifically, in pre-dialysis patients, most of the interventional studies could show only stabilization of renal function [42–45], whereas in a single interventional study, a positive effect of exercise on the progression of CKD was shown [46]. In this particular study, resistance training exercises together with low-protein diet were able to counterbalance protein catabolism in patients with renal failure, resulting in a significant decrease in inflammatory markers, improvement in muscle strength, and an improvement of eGFR.

A special group of patients with CKD is those on chronic hemodialysis. In this group, consistent evidence shows that aerobic exercise increases physical fitness, muscle strength, and quality of life [33, 47]. Moreover, in the absence of RCTs, cohort data show that dialysis patients, who engaged in more frequent exercise regimens, had a reduced mortality rate compared with their less active peers [48].

Kidney transplant recipients also benefit from participating in exercise. Studies have demonstrated improvements in muscle strength, exercise capacity, heart rate variability, and quality of life when patients engaged in regular aerobic or combined aerobic and resistance exercises [49–51]. However, these studies could not demonstrate benefits with respect to renal function, possibly because of eGFR in normal or near-normal range and a relatively short follow-up.

## Physical Activity in Preventing Initiation and Progression of DKD in Type 1 Diabetes

Individuals with diabetes and proteinuria typically develop progressive renal failure. Hence, possible beneficial effects of physical activity on kidney disease initiation and progression in this population are of great clinical importance.

While animal models investigating the effect of aerobic training have shown reduced progression of nephropathy in rats with type 1 diabetes [52], understanding of the effects of exercise among patients with diabetes from interventional studies remains limited. In patients with type 2 diabetes, strenuous exercise was shown to worsen albuminuria immediately after the activity [53]. Thirty minutes after exercise, urinary albumin excretion rate is increased, at least partly influenced by the increased angiotensin-2 level and enhanced glomerular membrane permeability. In addition, lactic acid, that is produced during strenuous exercise, is filtered by the glomeruli, enters the tubular lumen, and inhibits reabsorption of proteins at the proximal tubule, such as beta2-microglobulin, thus leading to tubular proteinuria [54].

Large randomized controlled trials studying the effects of physical activity on nephropathy in patients with type 1 diabetes do not exist. The first available data on exercise effects on microvascular complications in type 1 diabetes came from the Pittsburgh study [55]. The study showed that current physical activity as well as physical activity that the patients undertook during adolescence was inversely associated with the risk of diabetic nephropathy and neuropathy in men, but not in women. Unsurprisingly, patients with nephropathy undertook less physical activity, probably as a consequence of adverse effects of renal disease. Interestingly, in the FinnDiane cross-sectional study, even patients with microalbuminuria were less physically active compared with patients with normoalbuminuria [56].

Prospective data from two large cohorts that evaluated the effects of physical activity on nephropathy are available. The first one is the DCCT cohort [57]. Since the study included newly diagnosed type 1 diabetes patients, the number of renal events was small and perhaps that was the reason why the study did not find any association between physical activity and nephropathy development. Nevertheless, the authors concluded that since they could not detect any harm imposed by exercise on renal disease, exercise should be encouraged in management of type 1 diabetes.

The other large set of data comes from our FinnDiane study that includes patients with type 1 diabetes at various disease stages. Its primary aim is to identify clinical, biochemical, and genetic factors that predispose to diabetic nephropathy and other chronic diabetic complications. In the study that investigated the effect of exercise on diabetic nephropathy, we included 1424 extensively characterized patients with diabetes duration of more than 20 years and followed their progression in albumin excretion rate or development of ESRD through a mean follow-up time 6.4 ± 3.1 years [58]. At baseline, the leisure time physical activity level was assessed using a comprehensive validated self-report questionnaire. With the help of this questionnaire, 4 components of physical activity were assessed: total amount, intensity, frequency, and duration of physical activity. The study was able to demonstrate for the first time in a prospective setting that physical activity is associated with the initiation (development of de novo microalbuminuria) as well as the progression of diabetic nephropathy. Moreover, the data showed that it was not the total amount of physical activity that was important, but rather its intensity. Namely, the greater the intensity of exercise, the lower was the risk of occurrence or progression of diabetic nephropathy. The beneficial association of moderate and high intensity physical activity with the progression of renal disease was unaffected by the diabetes duration, age at diabetes onset, sex, or smoking. However, the strength of the association was attenuated after controlling for confounding factors such as HbA_1c_, BMI, blood pressure, and triacylglycerol [58]. In addition, albeit the study was longitudinal, it was observational by design; therefore, the possibility of reverse causality cannot be completely excluded. Consequently, we cannot exclude the possibility that factors, associated with the ability to exercise, but not physical activity per se, were associated with lower risk of adverse renal outcomes.

Of note, recent genetic data from three large case-control cohorts confirm a causal role of obesity in DKD using a Mendelian randomization analysis [59]. Therefore, physical activity could have positive effects on DKD progression also through its effects on reducing obesity.

### Physical Activity Effects on Cardiovascular Events and Mortality in Type 1 Diabetes

From the FinnDiane data, we were also able to show that frequent and intensive physical activity reduces cardiovascular events in type 1 diabetes [60•]. From the 2074 patients without prevalent cardiovascular disease included, aged 38.8 ± 12.4 years, with a diabetes duration of 21.7 ± 12.4 years, a total of 206 individuals experienced a cardiovascular event during a 10-year follow-up. We were able to show that the individuals who participated in low intensity levels of physical activity had a 59% greater relative risk of incident cardiovascular events compared with those who practiced high intensity physical activity, even after adjustment for sex, diabetes duration, age of diabetes onset, and the presence of diabetic nephropathy.

Furthermore, our group has recently investigated whether exercise amount or intensity influences the risk of premature mortality in patients with type 1 diabetes [61]. We included 2639 patients, 40.1 ± 12.6 years old, with 23.3 ± 12.8 years of diabetes duration. A total of 270 deaths occurred over a period of 11.4 ± 3.5 years. The total amount of self-reported physical activity at baseline was significantly associated with the mortality at follow-up, even after adjusting for sex, smoking, diabetes duration, age of diabetes onset, the presence of diabetic nephropathy, HbA_1c_, triglycerides, systolic blood pressure, and BMI. In addition, the relative risk of premature mortality rate was almost doubled in face of low versus moderate/high level of either of exercise components, including intensity, duration or frequency, even after adjustment for sex, diabetes duration, age of diabetes onset, smoking, and nephropathy presence [61]. Patients with CKD, defined in this study as eGFR below 60 ml/min/1.73m^2^, experienced similar beneficial effects of physical activity on mortality, although when adjusted for the confounders specifically the total amount of leisure time physical activity and exercise frequency were independently associated with all-cause mortality [61]. Altogether, our results suggest that exercise, in particular a high frequency and high intensity exercise, may reduce the risk of diabetic kidney disease, cardiovascular disease, and mortality in type 1 diabetes.

## Contraindications to Physical Activity

While engaging in physical activity may exert multiple beneficial effects on well-being over time, sedentary patients with diabetes should be educated on potential adverse events associated with the initiation of an exercise program. For example, the risk of hypoglycemia may be increased during and after exercise. Therefore, in preparation for exercise, individuals should be aware of their blood glucose concentrations, have their blood glucose monitoring equipment, and snacks in cases of hypoglycemia available. Aerobic exercise should be started only after the glucose concentration is above 7 mmol/l (126 mg/dl); however, anaerobic exercise can be started after glucose levels are above 5 mmol/l (90 mg/dl). If the glucose concentration is above 15 mmol/l (270 mg/dl), exercise could promote further hyperglycemia, especially in case of ketonemia [62•].

In addition, in individuals with longstanding diabetes and high HbA_1c_, vigorous exercise or activities involving heavy weight lifting or competitive endurance events are contraindicated. It is particularly true if the individual is suffering from proliferative diabetic retinopathy or severe non-proliferative diabetic retinopathy because of the risk of triggering vitreous hemorrhage or retinal detachment [1]. In addition, patients with cardiac autonomic neuropathy and renal failure should undergo clinical evaluation before intensifying exercise regimen [62•]. Patients on beta-blockers should be informed that adrenergic symptoms of hypoglycemia may be blunted during exercise [63]. Also, in patients with neuropathy, careful feet inspection is needed to prevent foot ulceration.

## Conclusions

In individuals with type 1 diabetes at risk of DKD or with established DKD, regular moderate-to-vigorous physical activity is associated with reduced incidence and progression of kidney disease, as well as reduced risk of cardiovascular events and mortality. Since structured physical activity has great potential to enhance health and quality of life at all stages of chronic kidney disease [33], exercise advice and physical activity assessment should become a routine integral part of the patient-centered treatment strategy also in type 1 diabetes.

Even though guidelines regarding the optimal type, frequency, intensity, and duration of physical activity for preventing DKD or DKD progression have yet to be formalized, it seems reasonable, in patients without contraindications, to follow the general advice of at least 150 min of moderate-to-vigorous aerobic exercise per week, combined with resistance exercises two to three times per week [1]. Promoting exercise in individuals with type 1 diabetes needs awareness and acknowledgment of all the potential barriers to physical activity, with special focus on improving patient education and autonomy around exercise self-management.
